# Development of Shuttle Vectors for Transformation of Diverse *Rickettsia* Species

**DOI:** 10.1371/journal.pone.0029511

**Published:** 2011-12-21

**Authors:** Nicole Y. Burkhardt, Gerald D. Baldridge, Phillip C. Williamson, Peggy M. Billingsley, Chan C. Heu, Roderick F. Felsheim, Timothy J. Kurtti, Ulrike G. Munderloh

**Affiliations:** 1 Department of Entomology, University of Minnesota, St. Paul, Minnesota, United States of America; 2 Department of Forensic and Investigative Genetics, University of North Texas Health Science Center, Fort Worth, Texas, United States of Ameirca; Louisiana State University and A & M College, United States of America

## Abstract

Plasmids have been identified in most species of *Rickettsia* examined, with some species maintaining multiple different plasmids. Three distinct plasmids were demonstrated in *Rickettsia amblyommii* AaR/SC by Southern analysis using plasmid specific probes. Copy numbers of pRAM18, pRAM23 and pRAM32 per chromosome in AaR/SC were estimated by real-time PCR to be 2.0, 1.9 and 1.3 respectively. Cloning and sequencing of *R. amblyommii* AaR/SC plasmids provided an opportunity to develop shuttle vectors for transformation of rickettsiae. A selection cassette encoding rifampin resistance and a fluorescent marker was inserted into pRAM18 yielding a 27.6 kbp recombinant plasmid, pRAM18/Rif/GFPuv. Electroporation of *Rickettsia parkeri* and *Rickettsia bellii* with pRAM18/Rif/GFPuv yielded GFPuv-expressing rickettsiae within 2 weeks. Smaller vectors, pRAM18dRG, pRAM18dRGA and pRAM32dRGA each bearing the same selection cassette, were made by moving the *parA* and *dnaA-*like genes from pRAM18 or pRAM32 into a vector backbone. *R. bellii* maintained the highest numbers of pRAM18dRGA (13.3 – 28.1 copies), and *R. parkeri*, *Rickettsia monacensis* and *Rickettsia montanensis* contained 9.9, 5.5 and 7.5 copies respectively. The same species transformed with pRAM32dRGA maintained 2.6, 2.5, 3.2 and 3.6 copies. pRM, the plasmid native to *R. monacensis*, was still present in shuttle vector transformed *R. monacensis* at a level similar to that found in wild type *R. monacensis* after 15 subcultures. Stable transformation of diverse rickettsiae was achieved with a shuttle vector system based on *R. amblyommii* plasmids pRAM18 and pRAM32, providing a new research tool that will greatly facilitate genetic and biological studies of rickettsiae.

## Introduction

The genus *Rickettsia* comprises obligate intracellular, gram-negative alphaproteobacteria associated with arthropods that feed on vertebrates and plants. Rickettsiae have been notoriously resistant to genetic manipulation and analysis but discovery of plasmids within their reduced genomes [1], [2], [3], [4] suggests possible development of shuttle vectors as an alternative to transposon-based transformation of rickettsiae [5], [6]. Sequenced and annotated rickettsial plasmids carry genes encoding potential environmental and host adaptive proteins such as small heat shock proteins and patatin, a putative virulence factor [1], [4]. Plasmids may also serve as repositories for horizontally acquired genes that enhance rickettsial competitiveness in the intracellular arena [7]. Discovery of multiple distinct plasmids in *Rickettsia amblyommii*, each carrying different *parA* genes that presumably facilitate their coexistence by avoiding plasmid incompatibilities [3], [7] was a seminal finding. The ability of rickettsiae to maintain multiple plasmids carrying horizontally acquired genes suggested that rickettsial plasmids could be used to develop shuttle vectors that would be maintained during long-term cultivation and enable analysis of gene function in rickettsiae.

We previously cloned and sequenced the *R. amblyommii* AaR/SC plasmids pRAM18 and pRAM23 [4] and now report the sequence of a third plasmid, pRAM32. The goal of this research was to construct shuttle vectors for the transformation of a range of *Rickettsia* species. We tested efficacy of shuttle vectors based on pRAM18 and pRAM32 in the transformation of plasmid-free rickettsiae (*Rickettsia parkeri, Rickettsia bellii,* and *Rickettsia montanensis*) and in *Rickettsia monacensis* whose native plasmid encodes a different *parA* gene. We achieved effective and stable transformation of all four species. Development of these shuttle vectors overcomes long-standing barriers to genetic manipulation of rickettsiae and will facilitate analysis of gene function in rickettsiae without unintentional disruption of native chromosomal or plasmid genes by transposons.

## Results

### Cloning and sequencing the pRAM32 plasmid

While cloning pRAM18 and pRAM23 from a genomic library of *R. amblyommii* AaR/SC, we obtained 15 kbp of a provisional third plasmid [4]. We PCR-amplified the remainder of the plasmid using end sequence complementary primers and sequenced the overlapping 18,408 bp amplicon. The third plasmid, pRAM32, was determined to be 31,972 bp in length with a G/C content of 34%. Twenty-two genes or pseudogenes were predicted by comparison of pRAM32 nucleotide or translated sequences with sequences in GenBank using blastn or blastx (NCBI). The degree of congruence between pRAM32 and other known rickettsial plasmids is low. Approximately 18% of its sequence has 74% or higher homology to pRAM18 and 22% of pRAM32 displays 79% or greater homology to pRAM23 and pRF (*Rickettsia felis*) sequences. Regions of similarity include genes encoding the DnaA-like replication initiator protein, transposases and the conjugal transfer protein TraA_Ti. The pRAM32 **dnaA-like gene has homologs on other rickettsial plasmids but its C-terminal domain is separated from the rest of the gene by 2,027 bp of sequence containing a transposase gene and a split transposase gene. Other features of interest in pRAM32 include a patatin-like phospholipase gene implicated in virulence [8], a 1,870 bp sequence with homology to a cluster of three transposase genes that recurs six times in the *R. felis* genome, a *traW* homolog encoding a type F conjugative transfer system protein and a pseudogene similar to *traU* encoding a conjugal DNA transfer protein on REIS, the rickettsial endosymbiont of *Ixodes scapularis*, the black-legged tick.

### Differentiating the *R. amblyommii* AaR/SC plasmids

**Figure 1:**
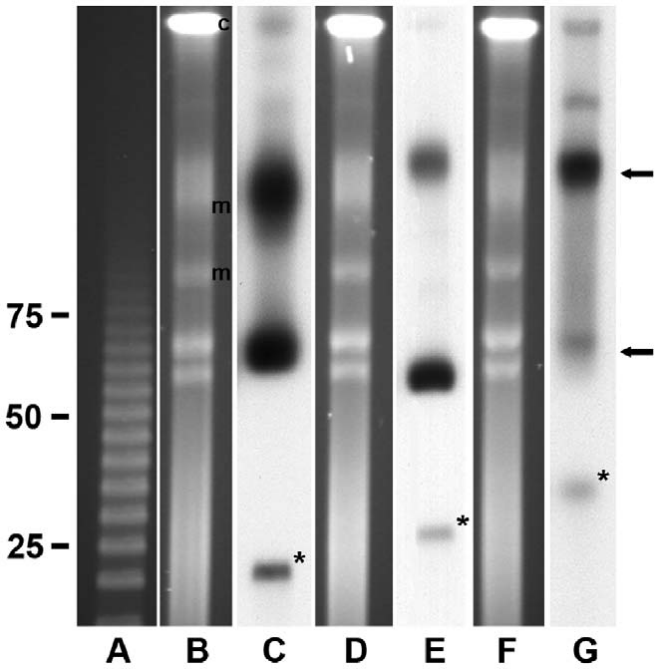
Pulsed-field gel electrophoresis and Southern analysis of the three *R. amblyommii* AaR/SC plasmids. (A) 5 kbp ladder. (B, D and F) Replicate panels of AaR/SC. (C) Southern analysis of gel from panel B hybridized with digoxigenin-labeled DNA Invertase probe, specific for pRAM18. (E) Southern analysis of gel from panel D hybridized with digoxigenin-labeled *hsp2* probe, specific for pRAM23. (G) Southern analysis of gel from panel F hybridized with digoxigenin-labeled *recD* probe, specific for pRAM32. Asterisks mark the putative linear monomer form of each plasmid and arrows indicate their predominant conformational isomers. Relative positions of ISE6 host cell mitochondrial, m, and chromosomal, c, DNAs are indicated to the right of the gel in panel B. Linear DNA marker positions in kbp are noted to the left of panel A.

Purified AaR/SC was embedded in agarose, lysed, divided into three lanes and separated by pulsed-field gel electrophoresis (PFGE) (Figure 1 panels B, D and F). Southern analysis was performed to illustrate the existence of three distinct plasmids by hybridization with one of three digoxigenin-labeled probes: a DNA Invertase gene probe specific for pRAM18, an *hsp2* probe specific for pRAM23, or a *recD* probe specific for pRAM32. The probes hybridized in distinctly different patterns exhibiting three main forms for each of the plasmids (Figure 1 panels C, E and G). The smallest and least abundant plasmid forms were putative linear monomers that were not visible on SYBR Green (Lonza, Rockland, ME) –stained gels but hybridized at approximately 22 kbp, 27 kbp and 34 kbp (indicated in Figure 1 with asterisks on panels C, E and G) relative to linear markers (panel A). The putative supercoiled forms of pRAM18 and pRAM32 co-migrated between the 65 and 70 kbp markers, while the putative pRAM23 supercoiled form migrated between the 55 and 60 kbp markers. This form of the plasmids appeared as a doublet on the SYBR Green-stained pulsed-field gels. Additional plasmid isomers approximately twice the size of the supercoiled forms migrated between the chromosomal and host cell mitochondrial DNAs. An uncharacterized fourth form was present in pRAM32 but absent from pRAM18 and pRAM23 while the abundance of supercoiled pRAM32 was quite low relative to supercoiled pRAM18 and pRAM23.

### Copy number of native plasmids in AaR/SC

Real time PCR primers were designed for single copy genes identified on each of the three plasmids. The relative ratio of *spoT*, pRAM23 *parA* or pRAM32 *parA* to chromosome-encoded *gltA* gave relative copy number values of 2.0, 1.9 and 1.3 for pRAM18, pRAM23 and pRAM32, respectively.

### Structure of pRAM18 and pRAM32 based shuttle vectors

**Figure 2:**
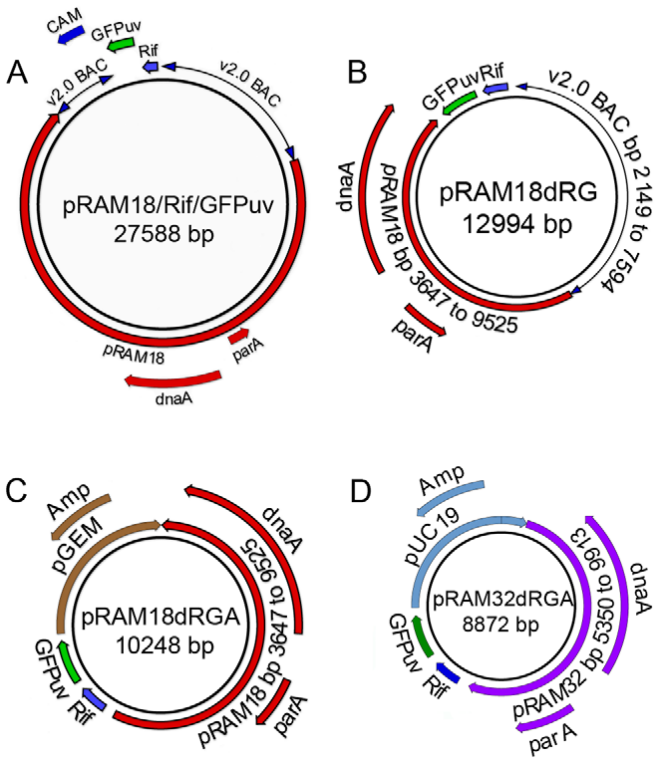
Maps of pRAM18 and pRAM32 derived shuttle vectors.

A 27.6 kbp recombinant plasmid pRAM18/Rif/GFPuv (Figure 2A) was used to derive two smaller shuttle vectors pRAM18dRG (Figure 2B) and pRAM18dRGA (Figure 2C). Portions of pRAM32 were used to construct the vector pRAM32dRGA (Figure 2D). All shuttle vectors were constructed to include the *parA* and *dnaA*-like genes and intervening sequences, and a selection cassette encoding rifampin resistance and a GFPuv fluorescent marker.

### Transformation of multiple *Rickettsia* species with shuttle vector constructs

**Figure 3:**
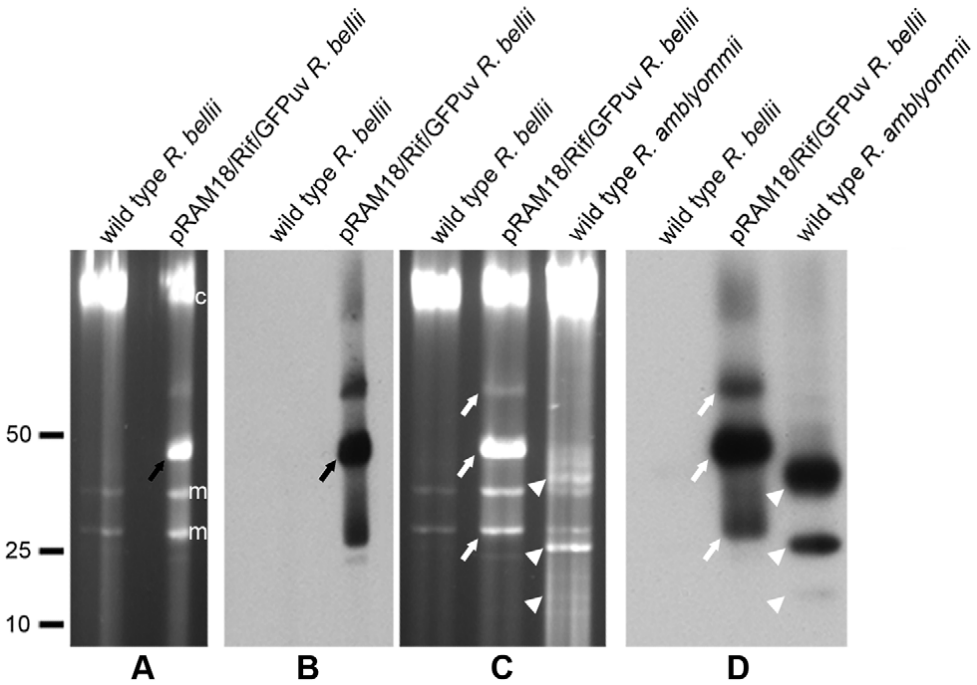
Pulsed-field gel electrophoresis and Southern analysis of pRAM18/Rif/GFPuv-transformed *R. bellii* RML 369-C. (A) PFGE gel and (B) Southern analysis of panel A hybridized with digoxigenin-labeled *gfp_uv_* probe showing the absence of plasmid in wild type *R. bellii* and the presence of plasmid in pRAM18/Rif/GFPuv-transformed *R. bellii*. Black arrows show the predominant form of pRAM18/Rif/GFPuv in transformed *R. bellii*. (C) PFGE and (D) Southern analysis of panel C hybridized with digoxigenin-labeled DNA Invertase probe, specific for pRAM18 plasmid. White arrows show conformational forms of pRAM18/Rif/GFPuv in transformed *R. bellii*, the smallest band (putative linear monomer) migrating as expected at 27,588 bp. Arrowheads show predominant forms of pRAM18 in wild type *R. amblyommii* the smallest of which migrates as expected at 18,433 bp. Relative positions of ISE6 host cell mitochondrial, m, and chromosomal, c, DNAs are indicated to the right of panel A. Linear DNA marker positions in kbp are noted to the left of panel A.

**Figure 4:**
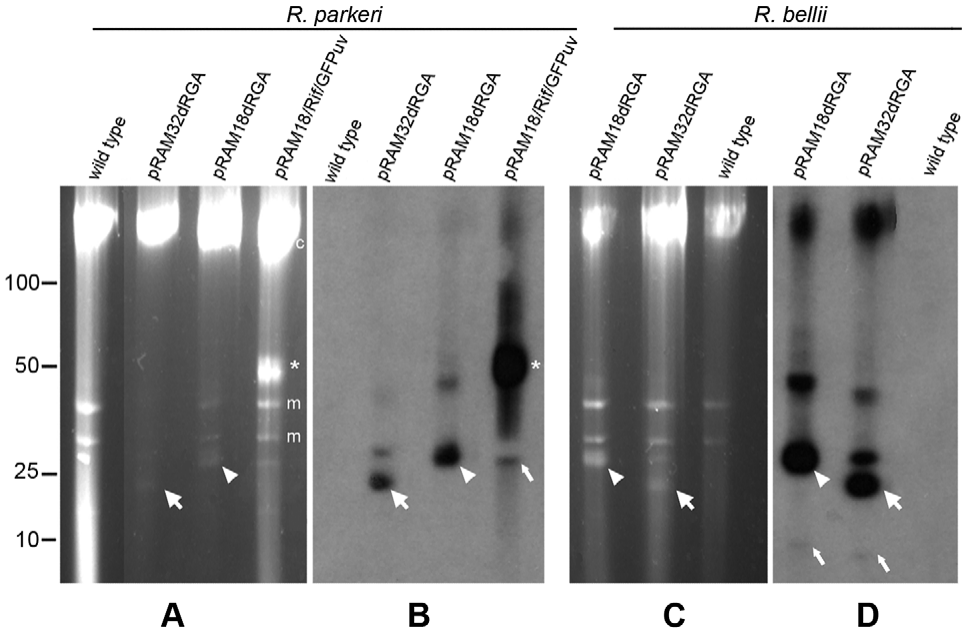
Plasmids in shuttle vector-transformed *R. parkeri* Oktibbeha and *R. bellii* RML369-C. (A and C) A single PFGE gel divided into two panels showing the presence of plasmids in pRAM18dRGA and pRAM32dRGA- transformed *R. parkeri* and *R. bellii*. Full-length pRAM18/Rif/GFPuv–transformed *R. parkeri* is included for comparison. (B and D) Southern analysis of panels A and C respectively, hybridized with a digoxigenin-labeled *gfp_uv_* probe confirming the presence of plasmids pRAM18dRGA and pRAM32dRGA in transformed *R. parkeri* and *R. bellii*. Asterisks, arrowheads and wide arrows indicate the predominant putative supercoiled form of pRAM18/Rif/GFPuv, pRAM18dRGA and pRAM32dRGA respectively, and the narrow arrows indicate putative linear monomeric plasmid. Relative positions of ISE6 host cell mitochondrial, m, and chromosomal, c, DNAs are indicated to the right of panel A. Linear DNA marker positions in kbp are noted to the left of panel A.

Table S1 lists rickettsiae species that were tested. Electroporation of *R. parkeri* Oktibbeha and *R*. *bellii* RML 369-C with the 27.6 kbp recombinant plasmid pRAM18/Rif/GFPuv (Figure 2A) and subsequent selection with rifampin yielded GFPuv-expressing rickettsiae in two weeks. A pulsed-field gel showed the presence of pRAM18/Rif/GFPuv in the *R. bellii* transformant, visible as a SYBR Green-stained DNA band migrating slightly below the 50 kbp linear marker (Figure 3A black arrow), and its absence in the wild type *R. bellii* lane. A Southern blot hybridized with *gfp*_*uv*_ confirmed the presence of pRAM18/Rif/GFPuv plasmid (Figure 3B). A second panel of the same pulsed-field gel demonstrated the presence of multiple plasmids in wild type *R. amblyommii* sample (Figure 3C, arrowheads), and the Southern blot hybridized with pRAM18-specific DNA Invertase probe showed co-localization of pRAM18 DNA Invertase with *gfp*_*uv*_ (Figure 3D) and identified native pRAM18 (18,422 bp) migrating in the expected pattern (Figure 3D, arrowheads) as compared to recombinant pRAM18/Rif/GFPuv (27,588 bp) (white arrows). PFGE and Southern analysis also confirmed the presence of pRAM18/Rif/GFPuv in transformed *R. parkeri* (Figure 4A and B; asterisks mark the predominant form of pRAM18/Rif/GFPuv).

In order to facilitate transformation, smaller shuttle vector constructs were derived from pRAM18/Rif/GFPuv (pRAM18dRG and pRAM18dRGA) and pRAM32 (pRAM32dRGA) (Figure 2B, C and D respectively). Plasmid-free *R. parkeri* Oktibbeha and *R. bellii* RML 369-C were transformed with pRAM18dRGA and pRAM32dRGA. GFPuv-expressing rickettsiae were detected in *R. parkeri* after 5 and 7 days, and in *R. bellii* after 10 and 9 days, respectively. The plasmids were visualized by PFGE (Figure 4A and C), and Southern blots hybridized with a *gfp*_*uv*_ probe (Figure 4B and D) indicated that the majority of the plasmids existed in the putative supercoiled state (wide arrows, arrowheads and asterisks) with very little of the linear monomer present (narrow arrows).

**Figure 5:**
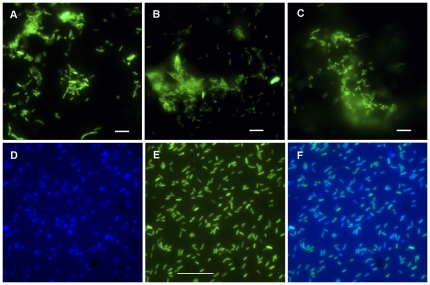
GFPuv fluorescent *R. montanensis* M5/6 transformed using shuttle vectors derived from *R. amblyommii* plasmids. (A – C) *R. montanensis* transformed with shuttle vectors: (A) pRAM18dRG; (B) pRAM18dRGA; or (C) pRAM32dRGA. Bar = 2 µm. Rickettsiae visualized using a fluorescein isothiocyanate (FITC) filter. (D – F) Cell-free pRAM18dRGA-transformed *R. montanensis* stained with DAPI and imaged on an Upright Nikon Eclipse E400 microscope with (D) DAPI or (E) FITC filters. (F) A composite image of panels D and E. Bar = 10 µm.

**Figure 6:**
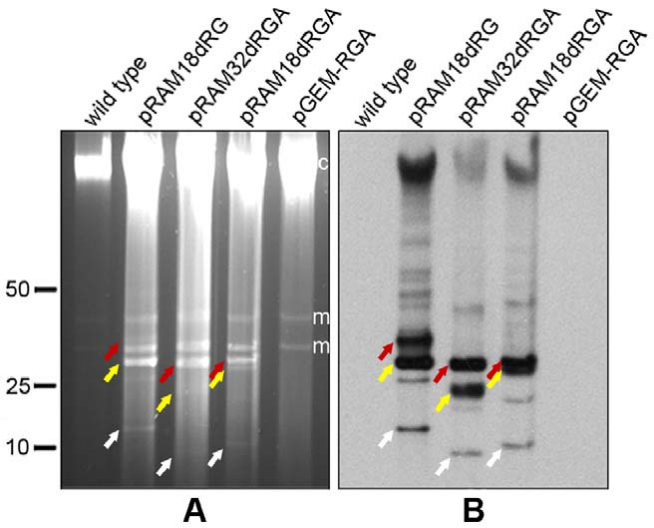
Presence of plasmids in shuttle vector-transformed *R. montanensis*. (A) Pulsed-field gel electrophoresis and (B) Southern analysis of the same gel hybridized with digoxigenin-labeled *gfp_uv_* probe showing the presence of plasmids in pRAM18dRG, pRAM32dRGA and pRAM18dRGA- transformed *R. montanensis*. Putative DNA conformational identities are indicated by the white (linear monomeric plasmid), yellow (supercoiled) and red (mixed circular and linear dimeric) arrows. Relative positions of ISE6 host cell mitochondrial, m, and chromosomal, c, DNAs are indicated to the right of the gel in panel A. Linear DNA marker positions in kbp are noted to the left of panel A.

*Rickettsia montanensis* M5/6, which also lacks a native plasmid, was successfully transformed with all three of the derived constructs, resulting in brightly fluorescent, GFPuv-expressing rickettsiae 20 days after electroporation (Figure 5 panels A, B and C). Analysis of *R. montanensis* pRAM18dRGA transformants stained with 4′,6-diamidino-2-phenylindole (DAPI) (VECTASHIELD, Vector Laboratories, Burlingame, CA) and examined by epifluorescence microscopy using DAPI or FITC filters showed over 98% congruence between rickettsia positive for DNA and rickettsia positive for GFPuv (Figure 5 panels D, E, and F), indicating that the rickettsial population contains few, if any, non-transformants. Wild type *R. montanensis* showed the presence of rickettsial DNA with DAPI but displayed no GFPuv fluorescence (data not shown). PFGE (Figure 6A) and Southern analysis (Figure 6B) of the *R. montanensis* shuttle vector transformants with a digoxigenin-labeled *gfp*_*uv*_ probe demonstrated that the relative positions of the plasmid forms were congruent with the expected sizes (pRAM18dRG: 12,994 bp, pRAM32dRGA: 8,872 bp and pRAM18dRGA: 10,248 bp) (Figure 6A and 6B, white arrows).

**Figure 7:**
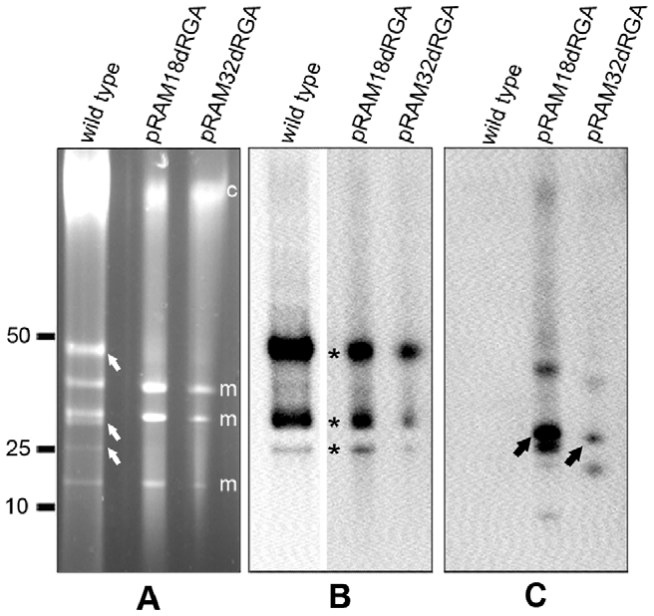
Pulsed-field gel electrophoresis and Southern analysis of *R. monacensis* transformants. (A) PFGE of wild type and shuttle vector-transformed *R. monacensis* showing the presence of native pRM in 3 forms, putative mixed circular and linear dimeric plasmid, supercoiled plasmid, and linear monomeric plasmid (white arrows). Relative positions of ISE6 host cell mitochondrial, m, and chromosomal, c, DNAs are indicated to the right of the gel. (B) Southern analysis of the same gel hybridized with digoxigenin-labeled *hsp2* probe showing the presence of native pRM in pRAM18dRGA and pRAM32dRGA-transformed *R. monacensis*. Asterisks denote positions of pRM forms. (C) Southern analysis of the same gel stripped and re-hybridized with digoxigenin-labeled *gfp_uv_* probe, confirming the presence of plasmids pRAM18dRGA and pRAM32dRGA in transformed *R. monacensis* and demonstrating coexistence of pRM with shuttle vectors. Black arrowheads mark the predominant forms of the shuttle vector plasmids. Linear DNA marker positions in kbp are noted to the left of panel A.

Two rickettsial species containing native plasmids were tested with pRAM18dRGA and pRAM32dRGA: *R. monacensis* and *R. amblyommii* AaR/SC. Both constructs yielded GFPuv-expressing *R. monacensis* 28 days after electroporation. A pulsed-field gel comparing wild type and transformed *R. monacensis* showed the presence of three main forms of the native plasmid, pRM, in the wild type DNA: putative mixed circular and linear dimeric plasmid, supercoiled plasmid, and linear monomeric plasmid (Figure 7A; arrows) [2]. These bands were not visible for the transformants on the SYBR green stained pulsed-field gel but a Southern blot hybridized with an *hsp2* probe, a gene present on pRM but not on pRAM18, showed that pRM was still present in the *R. monacensis* transformants populations (Figure 7B, asterisks). Hybridization of the same gel with a *gfp*_*uv*_-probe demonstrated that pRM co-existed with the shuttle vectors pRAM18dRGA and pRAM32dRGA (Figure 7C, black arrows). Electroporation of *R. amblyommii* AaR/SC with these constructs did not produce GFPuv-expressing rickettsiae, possibly due to exclusion of the shuttle vector by native plasmids containing identical incompatibility factors (see discussion).

### Recovery of plasmid from shuttle vector-transformed *R. montanensis*

Plasmids were successfully recovered from pRAM18dRGA and pRAM32dRGA-transformed *R. montanensis* and propagated in *Escherichia coli*. All endonuclease restriction digests of *E. coli*-recovered plasmids produced patterns as predicted.

### Shuttle vector copy numbers

Relative ratios of the shuttle vector-encoded *gfp*_*uv*_ and chromosome-encoded *gltA* genes were used to estimate copy number of the shuttle vectors in transformed rickettsiae (Table 1). Transformation with pRAM18dRGA resulted in higher copy numbers than did pRAM32dRGA. Although the copy number in rickettsiae transformed with pRAM32dRGA was relatively low, GFPuv-expressing rickettsiae were readily visible by fluorescence microscopy. pRAM18dRG was tested only in *R. montanensis* where it was maintained at almost the same copy number as pRAM18dRGA. Both the 27.6 kbp full-length plasmid pRAM18/Rif/GFPuv and its derivative pRAM18dRGA (10.2 kbp) yielded similar copy numbers in transformed *R. bellii* and *R. parkeri*. However, all electroporations with pRAM18dRGA in *R. bellii*, *R. parkeri*, *R. monacensis* and *R. montanensis* were successful, whereas pRAM18/Rif/GFPuv yielded transformants in *R. parkeri* and *R. bellii* but not in *R. montanensis* or *R. monacensis* (Table 1), indicating the derivative was more effective.

**Table 1:** Relative copy number of shuttle vectors in transformed *Rickettsia.*

Construct	*R. monacensis* Mean ± SD^a^	*R. montanensis* Mean ± SD	*R. bellii* Mean ± SD	*R. parkeri* Mean ± SD
pRAM18/Rif/GFPuv	NI^b^	NI	15.3±0.54	8.0±0.21
pRAM18dRGA	5.5±0.65	7.5±0.68	13.3±0.47 28.1±1.89^c^	9.9±1.63
pRAM18dRG	NT^d^	7.7±0.37	NT	NT
pRAM32dRGA	3.2±0.38	3.6±0.58	2.6±0.23	2.5±0.37

*^a^*Standard deviation.

*^b^*No transformant isolated.

*^c^*Results from 2 separate transformations of *R. bellii* with shuttle vector pRAM18dRGA.

*^d^*Not tested.

### Copy number of native pRM in pRAM18dRGA-transformed *R. monacensis*

The relative ratio of the pRM-encoded *hsp2* and chromosome-encoded *gltA* genes was used to estimate the copy number of native pRM in shuttle vector-transformed *R. monacensis* (Table 2). The average copy number of pRM in the first subcultures of pRAM18dRGA and pRAM32dRGA-transformed *R. monacensis* was comparable to the previously published average of 2.7 in wild-type *R. monacensis* [4]. The relative copy numbers of pRAM18dRGA ranged from about 3.6 to 5.5 during the course of 15 subcultures, the last 5 of which were done with and without rifampin selection in cultures maintained in parallel, indicating stable maintenance of both the native plasmid and the shuttle vectors during this period.

**Table 2:** Maintenance of native pRM in shuttle vector-transformed *R. monacensis*.

Rickettsial Culture	Relative Copy Number of pRM (*hsp2*/*gltA*)	Relative Copy Number of Shuttle Vector (*gfp_uv_*/*gltA*)
*R. monacensis*	2.7	NA
*R. monacensis* pRAM32dRGA transformant subculture 1	4.2	3.2
*R. monacensis* pRAM18dRGA transformant subculture 1	3.1	4.8
*R. monacensis* pRAM18dRGA transformant subculture 8	2.9	5.4
*R. monacensis* pRAM18dRGA transformant subculture 15	3.1	5.5
*R. monacensis* pRAM18dRGA transformant subculture 15 (No Rif for 5 subcultures)	2.5	3.6

### Expression of mCherry in a shuttle vector containing a multiple cloning site

**Figure 8:**
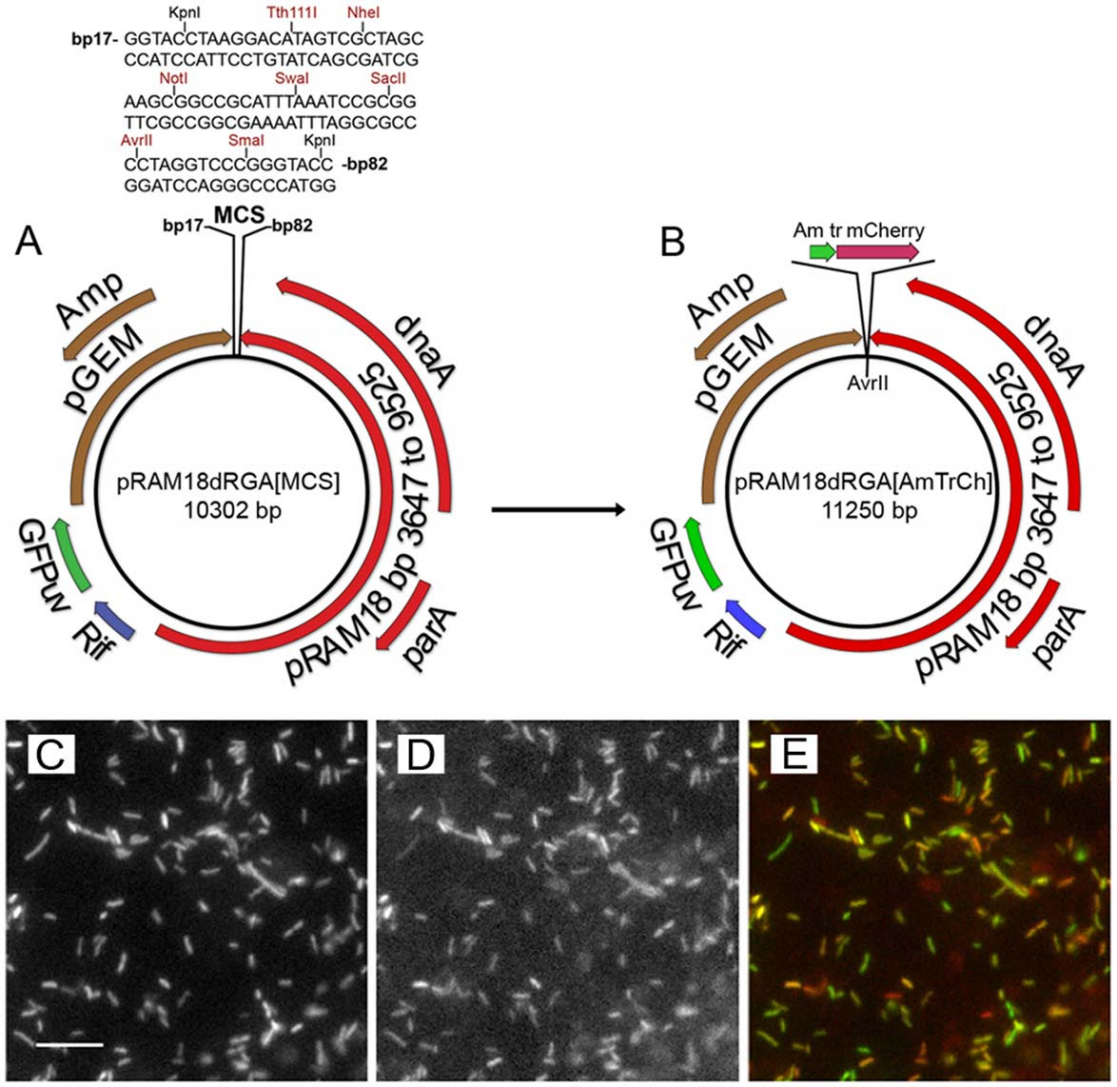
Transformation of *R. montanensis* with pRAM18dRGA[AmTrCh]. (A and B) Maps showing: (A) Restriction sites present in pRAM18dRGA[MCS]. (B) Insertion of AmTr/mCherry cassette into the MCS at the AvrII restriction site, yielding pRAM18dRGA[AmTrCh]. (C – E) Photomicrographs of cell free rickettsial suspensions centrifuged onto microscope slides and air dried showing: (C) GFPuv-positive *R. montanensis* transformed with pRAM18dRGA[AmTrCh], illuminated using the FITC filter. (D) Same microscopic field shown in C illuminated using the TRITC filter to reveal mCherry fluorescent positive rickettsiae. (E) Composite image made by merging images shown in C and D. Individual rickettsiae were examined on a TE2000-U Inverted microscope using epifluorescence illumination, with FITC and TRITC filter sets. Collected images of fluorescent rickettsiae from the red and green emission channels were processed in Image J. Bar = 10 µm.

A multiple cloning site (MCS) containing 7 restriction sites was inserted into pRAM18dRGA yielding pRAM18dRGA[MCS] (Figure 8A). A 948 bp expression cassette encoding mCherry red fluorescent protein under an *Anaplasma marginale* promoter [9], [10], [11]was inserted into the MCS to test the construct as a functional shuttle vector, yielding pRAM18dRGA[AmTrCh] (Figure 8B). *R. montanensis* transformants expressed both fluorescent proteins (Figure 8C, D and E), were Southern-blot-positive with digoxigenin-labeled *gfp*_*uv*_ and *mCherry* probes, and contained intact plasmids that were recovered in *E. coli*.

## Discussion

Methods for genetic transformation of rickettsiae have only recently been developed. Unlike bacteria that can be grown axenically, transformation of rickettsiae is inherently difficult to achieve due to their obligate intracellular existence. The need to physically remove rickettsiae from host cells before transformation while preserving subsequent infectivity poses an enormous challenge as electroporation requires their exposure to buffers lacking elements of the intracellular milieu required to sustain viability and infectivity. Current methods are limited to homologous recombination [12], [13], [14], [15] and transposase-mediated mutagenesis with TN5 transposon vectors [16], [17] and the Himar1 transposon system [5], [6]. These protocols make use of transformation vectors that can be generated in *E. coli* but do not replicate in rickettsiae where they are only transiently active. By contrast, plasmid systems for transformation of bacteria that do not require a eukaryotic host cell have been in use for decades and are well characterized. The ability of plasmids to replicate independently from chromosomal DNA has long been exploited to introduce genes of interest into extracellular bacteria. The recent discovery of native rickettsial plasmids has now provided a basis for development of successful plasmid-based transformation systems for rickettsiae. Rickettsial plasmids carry predicted *parA* and *dnaA*-like genes encoding proteins important for plasmid replication, maintenance and partitioning. The *parA* genes are quite diverse and display homology to phylogenetically distant bacterial groups suggesting that rickettsiae acquired plasmids by horizontal transfer from unrelated taxa [4], [7]. Most rickettsiae appear to have one plasmid but some such as *R. amblyommii* and REIS [4] harbor multiple plasmid types carrying different *parA* genes, suggesting that incompatibility groups are operational in rickettsiae.

Here, we report the development of shuttle vectors containing rickettsial plasmid maintenance genes and their use in transformation of rickettsiae. The vectors contain the *R. prowazekii arr-2* rifampin resistance cassette, *rpsLp-arr-2*_Rp_, for selection of transformants and a reporter gene, *gfp*_*uv*_, under control of the *R. rickettsii ompA* promoter [5], [16]. They also contain crucial *parA* and *dnaA*-like genes and intervening sequences from two low copy number *R. amblyommii* plasmids, pRAM18 and pRAM32, enabling autonomous replication of constructs containing foreign DNA in tested Rickettsia species. We have demonstrated the utility of shuttle vectors that include these regions from pRAM18 and pRAM32 and note that the *parA* - *dnaA* region from pRAM23 can also be used to construct a shuttle vector that functions in rickettsiae (unpublished data). Finally, we constructed the pRAM18dRGA[MCS] vector containing a multiple cloning site for facile insertion of genes of interest. As a proof of concept, we inserted a gene for the red fluorescent protein mCherry and obtained rickettsial transformants expressing both red and green fluorescent proteins. Maintenance of the shuttle vectors over serial passage in one transformed *Rickettsia* species harboring a native plasmid as well as in three species without native plasmids indicated that they will be of general utility for rickettsial transformation. However, we have been unable to transform wild type *R. amblyommii* with these vectors or *Rickettsia massiliae* with pRAM18dRGA. *R. massiliae* has a native plasmid, pRMA, carrying a *parA* gene homologous to that of pRAM18dRGA. Yet *R. massiliae* can be successfully transformed with a shuttle vector containing the *parA* - *dnaA*-like region of pRAM23 (unpublished data), in which the *parA* is not homologous to that of pRMA. These failures and successes point to the role of parA genes in maintaining compatible plasmid complements in rickettsiae.

In recipient rickettsiae the shuttle vectors were maintained at the low copy number phenotype of native plasmids for at least 15 serial transfers with continuous antibiotic selection and at least 5 serial transfers without antibiotic selection, while directing expression of fluorescent reporter and antibiotic resistance proteins at levels that permitted ready isolation and visualization of transformants. In contrast to earlier studies [12], we did not experience emergence of non-specific rifampin resistant spontaneous mutants.

The frequency/efficiency of transformation after electroporation of rickettsiae with shuttle vectors was much lower than that observed with E. coli, as judged by the time elapsed until microscopic detection of transformants, and proportion of host cells harboring transformants. Generally, 2 to 3 weeks transpired between electroporation and the appearance of transformants. The low number of transformants from an electroporation suspension containing approximately 10^8^ rickettsiae and 10^10^ plasmids indicates that either very few rickettsiae survived electroporation or they were not successfully transfected during electroporation. Nevertheless, these transformation rates are sufficiently reproducible to make the shuttle vectors a research tool that will greatly facilitate genetic and biological studies of the interactions of rickettsiae with host cells [18]. Shuttle vectors will enable analysis of rickettsial promoters or regulatory elements in their native host rather than in distantly related bacteria. They will be powerful tools for studying the interaction of rickettsiae with host cells in vivo and in vitro. Rickettsial transformants carrying plasmid constructs with selectable and reporter genes can be tested for occurrence and frequency of rickettsial conjugation. Although plasmids exist in most Rickettsia spp., natural genetic transfer systems such as conjugation remain to be demonstrated. Conjugation is generally a plasmid-encoded process and homologs of tra genes involved in conjugation are found on rickettsial plasmids and chromosomes. Evidence for formation of pili-like structures has been observed in R. felis [1], R. bellii [19] and R. massiliae [20] but direct evidence of conjugation resulting in DNA transfer from one rickettsia to another is lacking. The shuttle vectors also will enable genetic complementation of mutants generated naturally or by means such as transposon mutagenesis or homologous recombination. Complementation analyses using plasmids that do not introduce genomic “hits” will avoid inadvertent generation and subsequent analysis of the effects of new and unknown potential knockouts. Lastly, the site-directed mutagenesis studies that have been of great utility in studies of extracellular bacteria have largely remained out of reach to rickettsiologists, but a replicating rickettsial plasmid containing sequences homologous to target genes could potentially increase the efficiency of homologous recombination and thus enable such studies in rickettsiae.

## Materials and Methods

### Rickettsiae

Plasmid DNA for shuttle vector construction was obtained from *R. amblyommii* AaR/SC [3], [4]. The partial *ompA* (AF453408), *ompB* (JN378402), *gltA* (JN378401), and 17 kDa (JN378400) gene sequences of isolate AaR/SC are available at NCBI. *R. amblyommii* AaR/SC, *R. monacensis* (IrR/Munich), *R. montanensis* (M5/6), *R. bellii* (RML 369C), and *R. parkeri* (Oktibbeha) (Table S1) were used to test transformation efficiency of the shuttle vectors. Rickettsiae were grown in *Ixodes scapularis* cell line ISE6 in L-15B300 medium with NaHCO_3_ and HEPES buffer [21] in 25-cm^2^ tissue culture flasks. Rickettsiae from heavily infected cultures were purified from host cells as previously described [3].

### Cloning and sequencing of *R. amblyommii* AaR/SC plasmid pRAM32

The plasmid pRAM32 was cloned and sequenced as detailed in the Materials and Methods S1. Briefly, a fragment of pRAM32 previously cloned in pJazz (Lucigen, Middleton, WI) [4] was sequenced and primers complementary to the clone were used to obtain the remaining portion of the plasmid by PCR amplification of *R. amblyommii* AaR/SC genomic DNA. The resulting 18,408 bp amplicon was sequenced and annotated manually after submission to the NCBI Prokaryotic Genomes Automatic Annotation Pipeline (PGAAP). The nucleotide sequence of plasmid pRAM32 from *R. amblyommii* AaR/SC has been deposited in GenBank under the accession number CP002642.

### Construction of rickettsial shuttle vectors

Constructs were derived from two *R. amblyommii* AaR/SC plasmids: pRAM18 (Figure S1) and pRAM32 (Figure S2). Initially, a 27.6 kbp recombinant plasmid pRAM18/Rif/GFPuv (Figure 2A) was created by inserting a selection cassette into the entire pRAM18 plasmid cloned in the *E. coli* vector BACv2.0 (Lucigen) (Figure S1C). The selection cassette, derived from pMW1650 (kindly provided by D. H. Wood, University of South Alabama) [5], contained the *R. prowazekii arr-2* rifampin resistance gene (*rpsLp-arr-2_Rp_*, or Rif) and a gene coding for green fluorescent protein driven by the rickettsial *ompA* promoter (*ompAp*-GFPuv, or GFPuv). Smaller shuttle vectors, pRAM18dRG, pRAM18dRGA and pRAM32dRGA (Figure 2B, C and D), were constructed using the same selection cassette and the *parA* and *dnaA-*like genes from pRAM18 (Figure S1A) or pRAM32 (Figure S2A). Details are provided in Materials and Methods S1.

### Preparation of a shuttle vector containing a multiple cloning site

A multiple cloning site (MCS) was inserted into pRAM18dRGA yielding pRAM18dRGA[MCS] (Figure 8A). A 948 bp AmTr/mCherry cassette containing the *Anaplasma marginale* promoter *tr* [9], [10], [11] driving expression of *mCherry* encoding a red fluorescent protein was inserted into the MCS yielding pRAM18dRGA[AmTrCh] (Figure 8B). Construction of the vectors is described in Materials and Methods S1.

### Purification and transformation of rickettsiae

Rickettsiae purified [3] from one 25-cm^2^ flask were sufficient for two electroporation trials. Host cell-free rickettsiae were washed 3 times in 250 mM sucrose, resuspended in 250 mM sucrose and held on ice. Endotoxin-free plasmid DNA (1 µg) was placed in a chilled 0.1 cm gap electroporation cuvette (Bio-Rad, Hercules, CA) with 50 µl of rickettsiae in 250 mM sucrose and pulsed once at 1.8 kV, 200 ohms and 25 µF in a Gene Pulser II electroporation system (Bio-Rad). Electroporated rickettsiae were recovered in 1 ml of tick cell culture medium and transferred onto a confluent layer of ISE6 cells in a 25-cm^2^ flask. The volume of medium was brought up to 5 ml and cultures were incubated at 34^o^C for 24 h before adding 1–2 µg/ml of rifampin. Growth medium was changed every 3 or 4 days while maintaining continuous rifampin selection. Cultures were monitored weekly for presence of green fluorescent rickettsiae by epifluorescence microscopy using an inverted Nikon Diaphot or an upright Nikon Eclipse E400 (Nikon, Melville, NY) using Sapphire GFP or fluorescein isothiocyanate (FITC) filters, respectively.

### Examination of the homogeneity of shuttle vector-transformed rickettsiae

Purified wild type and pRAM18dRGA-transformed *R. montanensis* were affixed to slides by centrifugation using a Cytospin (Thermo Scientific, Pittsburgh, PA), air dried, and stained with VECTASHIELD mounting medium containing DAPI (Vector Laboratories). Slides were examined by epifluorescence microscopy using DAPI or FITC filters to determine the percentage of GFPuv-expressing transformants in the population.

### Preparation of rickettsial genomic DNA

Purified rickettsial pellets from one 5 ml culture were lysed with 300 µl Cell Lysis Solution and genomic DNA was purified with the Puregene Core A kit (Qiagen, Valencia, CA) according to the manufacturer's gram negative bacteria protocol.

### Recovery of plasmid from shuttle vector-transformed *R. montanensis*

224 ng and 177 ng of genomic DNA isolated from pRAM18dRGA and pRAM32dRGA-transformed *R. montanensis*, respectively, was used to transform *E. cloni* 10G elite electrocompetent cells as per manufacturer's protocol (Lucigen). Transformed *E. coli* were selected on YT/Rif/Amp plates, and plasmids isolated from clones were compared to the original plasmid by endonuclease restriction digest with SpeI, EcoRI, HindIII, PstI and XhoI (pRAM18dRGA clones) or SpeI, EcoRI, HindIII, PstI, XbaI and PvuII (pRA32dRGA clones).

### PFGE, Southern analysis and digoxigenin-labeled probe synthesis

Purified rickettsiae were separated on PFGE gels, transferred to Zeta Probe GT genomic membranes and hybridized with digoxigenin-labeled probes [2], [3] (Materials and Methods S1).

### Determination of shuttle vector and native plasmid copy numbers

Real-time quantitative PCR (qPCR) and the relative quantification method [22], [23] were used to determine shuttle vector and native plasmid copy number. Species-specific plasmids were used to generate standard curves with primers designed to target specific single copy genes (Tables S2 and S3) found on the shuttle vectors, native plasmids, and rickettsial chromosomal DNA (Materials and Methods S1).

### Stability of pRAM18dRGA in *R. monacensis* maintained in ISE6 cells

*R. monacensis* transformed with pRAM18dRGA was subcultured 1∶100 every 10 days for 15 serial transfers (50 µl of cell suspension from a heavily infected culture inoculated into an uninfected ISE6 culture) with continuous rifampin selection (0.8 µg/ml). At the first, eighth and fifteenth transfer the relative copy numbers of the shuttle vector (pRAM18dRGA) and native plasmid (pRM) was quantified. To determine the stability of pRAM18dRGA in transformed *R. monacensis* maintained in the absence of antibiotic selection, rifampin was withheld from the medium of a subculture at the tenth serial transfer. After 5 additional serial transfers in rifampin-free medium (15^th^ in vitro passage) the relative copy numbers of the shuttle vector (pRAM18dRGA) and native plasmid (pRM) were determined.

## Supporting Information

Figure S1 **The construction of shuttle vectors from** ***R. amblyommii*** **AaR/SC plasmid pRAM18.** The complete pRAM18 plasmid (A) was cloned from AaR/SC genomic DNA by digestion with SwaI and ligation into blunt pJazz OK. The resulting clone, pRAM18 pJazz (B), was digested with NotI to release pRAM18, which was then ligated into pSMART v2.0 BAC (C). The shuttle vector pRAM18/Rif/GFPuv (D) was assembled by ligating the ApaI-digested1.6 kbp PCR-amplified Rif/GFPuv cassette from pMW1650 (E) to ApaI-cut pRAM18 v2.0 BAC (C). The shuttle vector pRAM18dRG (F) was formed by ligating the NotI (partial v2.0 BAC with Rif and GFPuv expression) and BbvCI (the *parA* and the *dnaA*-like portion of pRAM18) fragments of pRAM18/Rif/GFPuv (D). The third pRAM18 shuttle vector, pRAM18dRGA (G), was constructed by cloning the BbvCI fragment of pRAM18/Rif/GFPuv (D) into SmaI-digested pGEM-RGA (H). pGEM-RGA was constructed by ligating the PstI-digested 1.6 kbp PCR-amplified Rif/GFPuv cassette from pMW1650 (E) to pGEM-3Z opened with PstI.
(TIF)

Figure S2 **Construction of a shuttle vector from** ***R. amblyommii*** **AaR/SC plasmid pRAM32.** A 15,043 bp fragment of the pRAM32 plasmid (A) was cloned from *R. amblyommii* AaR/SC genomic DNA by digestion with SwaI and ligation into blunt pJazz OK. The resulting clone, pRAM32 SwaI Frag 1 pJazz (B), was digested with XbaI and fragments were cloned into pUC19. PstI-cut pRAM32 XbaI frag 3 (C), the clone containing pRAM32 *dnaA*-like and *parA* genes, was ligated to the PstI-digested 1.6 kbp PCR-amplified Rif/GFPuv cassette from pMW1650 (D) to form the shuttle vector pRAM32dRGA (E).
(TIF)

Table S1 ***Rickettsia*** **species used to test rickettsial plasmid constructs.**
(DOC)

Table S2 **Single copy gene targets for determining relative copy number of native and shuttle vector plasmids.**
(DOC)

Table S3 **Real-time PCR Amplification Primers to Determine Relative Copy Number of Native and Shuttle Vector-Transformed** ***Rickettsia spp*** **.**
(DOC)

Materials and Methods S1 (DOC)
