# Supplementary figures and images for: Development of Shuttle Vectors for Transformation of Diverse Rickettsia Species
Source: PLoS One. 2011 Dec 21;6(12):e29511. doi: 10.1371/journal.pone.0029511 (PMC3244465; doi:10.1371/journal.pone.0029511)

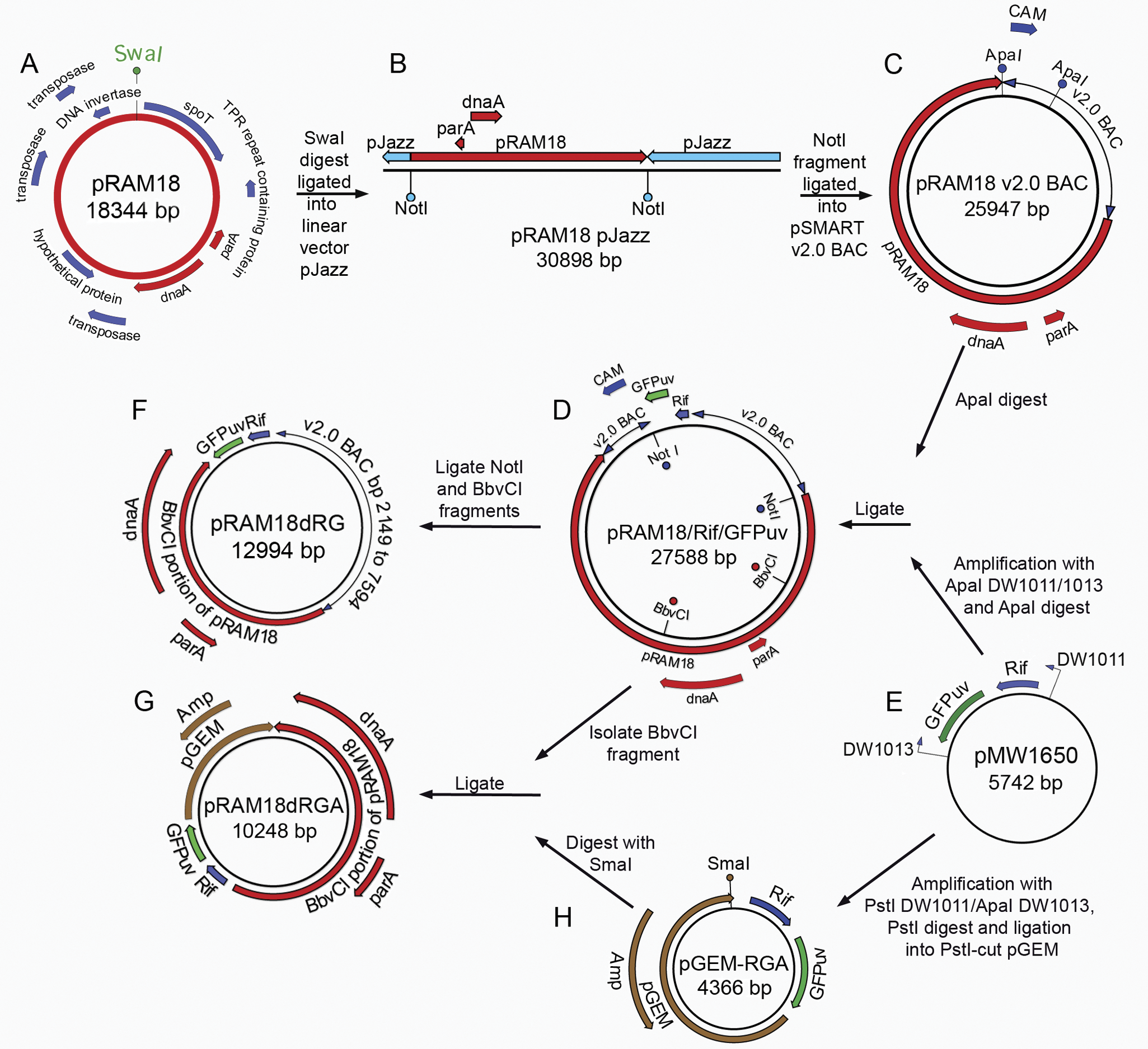

Supplement: Figure S1 — The construction of shuttle vectors from R. amblyommii AaR/SC plasmid pRAM18. The complete pRAM18 plasmid (A) was cloned from AaR/SC genomic DNA by digestion with SwaI and ligation into blunt pJazz OK. The resulting clone, pRAM18 pJazz (B), was digested with NotI to release pRAM18, which was then ligated into pSMART v2.0 BAC (C). The shuttle vector pRAM18/Rif/GFPuv (D) was assembled by ligating the ApaI-digested1.6 kbp PCR-amplified Rif/GFPuv cassette from pMW1650 (E) to ApaI-cut pRAM18 v2.0 BAC (C). The shuttle vector pRAM18dRG (F) was formed by ligating the NotI (partial v2.0 BAC with Rif and GFPuv expression) and BbvCI (the parA and the dnaA-like portion of pRAM18) fragments of pRAM18/Rif/GFPuv (D). The third pRAM18 shuttle vector, pRAM18dRGA (G), was constructed by cloning the BbvCI fragment of pRAM18/Rif/GFPuv (D) into SmaI-digested pGEM-RGA (H). pGEM-RGA was constructed by ligating the PstI-digested 1.6 kbp PCR-amplified Rif/GFPuv cassette from pMW1650 (E) to pGEM-3Z opened with PstI. (TIF) [file pone.0029511.s001.tif]

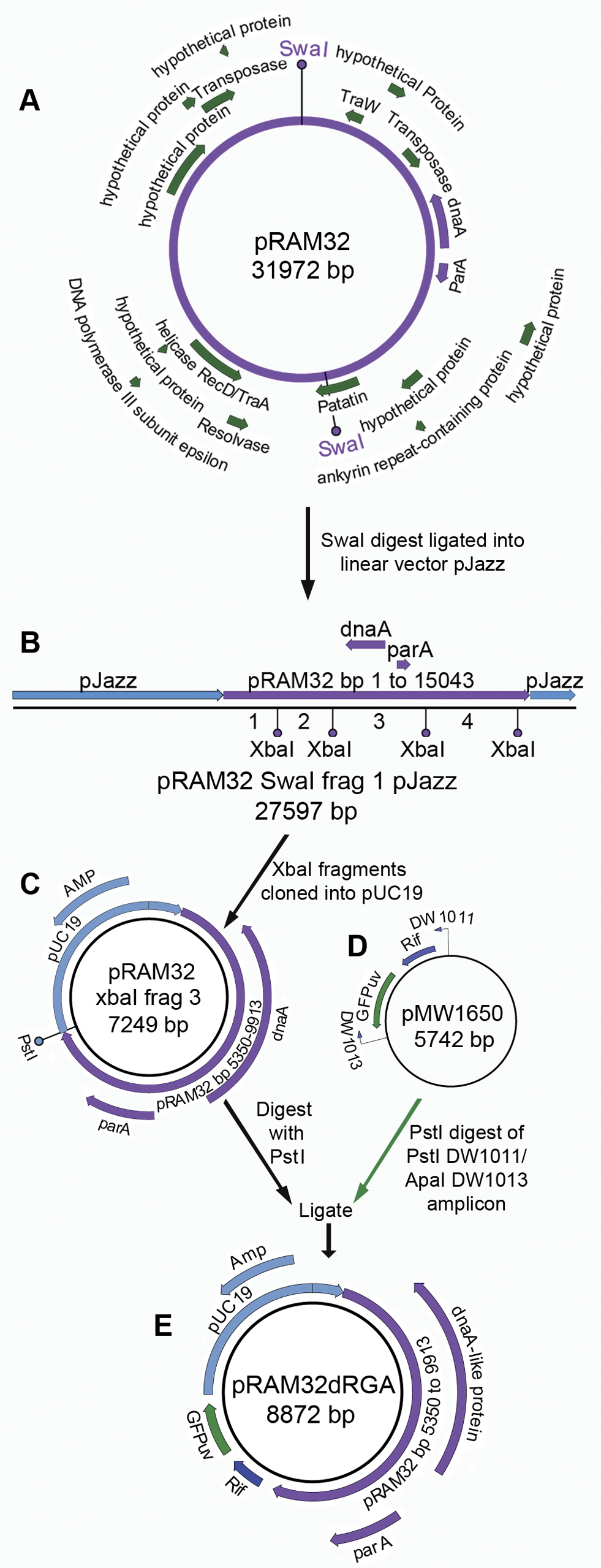

Supplement: Figure S2 — Construction of a shuttle vector from R. amblyommii AaR/SC plasmid pRAM32. A 15,043 bp fragment of the pRAM32 plasmid (A) was cloned from R. amblyommii AaR/SC genomic DNA by digestion with SwaI and ligation into blunt pJazz OK. The resulting clone, pRAM32 SwaI Frag 1 pJazz (B), was digested with XbaI and fragments were cloned into pUC19. PstI-cut pRAM32 XbaI frag 3 (C), the clone containing pRAM32 dnaA-like and parA genes, was ligated to the PstI-digested 1.6 kbp PCR-amplified Rif/GFPuv cassette from pMW1650 (D) to form the shuttle vector pRAM32dRGA (E). (TIF) [file pone.0029511.s002.tif]
