# Supplementary material for: Development of Shuttle Vectors for Transformation of Diverse Rickettsia Species
Source: PLoS One. 2011 Dec 21;6(12):e29511. doi: 10.1371/journal.pone.0029511 (PMC3244465; doi:10.1371/journal.pone.0029511)
Supplement: Table S1 — Rickettsia species used to test rickettsial plasmid constructs. (DOC) [file pone.0029511.s003.doc]

## Table S1. *Rickettsia* species used to test rickettsial plasmid constructs

| **Species** | **Isolate** | **Native Plasmids** | **Plasmid ID** | **Plasmid Size** | **Copy Number** |
| --- | --- | --- | --- | --- | --- |
| *R. amblyommii* | AaR/SC | 3 | pRAM18 | 18,344 bp | 2.0^a^ |
|  |  |  | pRAM23 | 22,852 bp | 1.9^b^ |
|  |  |  | pRAM32 | 31,972 bp | 1.3^c^ |
| *R. monacensis* | IrR/Munich | 1 | pRM | 23,486 bp | 2.7^d^ |
| *R. montanensis* | M5/6 | 0 | NA | NA | NA |
| *R. bellii* | RML 369C | 0 | NA | NA | NA |
| *R. parkeri* | Oktibbeha | 0 | NA | NA | NA |

NA=not applicable

^a^ based on *spoT*:*gltA* ratio

^b^ based on pRAM23*parA:gltA* ratio

^c^ based on pRAM32*parA*:*gltA* ratio

^d^ based on *hsp2:gltA* ratio
