# Supplementary material for: Development of Shuttle Vectors for Transformation of Diverse Rickettsia Species
Source: PLoS One. 2011 Dec 21;6(12):e29511. doi: 10.1371/journal.pone.0029511 (PMC3244465; doi:10.1371/journal.pone.0029511)
Supplement: Table S2 — Single copy gene targets for determining relative copy number of native and shuttle vector plasmids. (DOC) [file pone.0029511.s004.doc]

**Table S2. Single copy gene targets for determining relative copy number of native and shuttle vector plasmids**

| **Primer pair** | **Plasmid DNA for standard curve template** | **Target single copy gene** |
| --- | --- | --- |
| qCSF/qCSR-AcPa | Ac/Pa CS877/1273^*^ | Chromosomal *gltA* in *R. amblyommii* AaR/SC |
| qSpoTF2/qSpoTR1 | pRAM18 v2.0 BAC**^1^** | pRAM18 *spoT* in AaR/SC |
| AcPa-qHsp2F1/AcPa-qHsp2R1 | Ac/Pa Hsp2F3/R3* | pRAM23 *hsp2* in AaR/SC |
| pRAM23 qParAF/qParR | AaR/SC pRAM23 HpaI fragment in pJazz* | pRAM23 *parA* in AaR/SC |
| pRAM32 qParAF2/qParR2 | pRAM32 XbaI frag 3**^1^** | pRAM32 *parA* in AaR/SC |
| qGFPuvF/qGFPuvR | pMODompACAT/GFPuv 658**^2^** | Shuttle vector *gfp_uv_* in *R. monacensis, R. montanensis, R. parkeri* and *R. bellii* transformants |
| qCSF/qCSR | *R.massiliae* CS877/1273* | Chromosomal *gltA* in *R. parkeri*, *R. monacensis* and *R. montanensis* transformants |
| *R. bellii* qCSF/*R.bellii* qCSR | *R. bellii* CS877/1273 * | Chromosomal *gltA* in *R. bellii* transformants |
| qHsp2F/qHsp2R | *R. monacensis* Hsp2F3/R3* | Native pRM *hsp2* in *R. monacensis* transformants. |

*See (1) for construction of these plasmids

**^1^**This publication

**^2^**See (2) for construction of this plasmid

1. Baldridge GD*, et al.* (2010) Wide dispersal and possible mutliple origins of low-copy-number plasmids in *Rickettsia* species associated with blood-feeding arthropods. *Appl. Environ. Microbiol.* 76:1718-1731.

2. Baldridge GD, Burkhardt N, Herron MJ, Kurtti TJ, & Munderloh UG (2005) Analysis of fluorescent protein expression in transformants of Rickettsia monacensis, an obligate intracellular tick symbiont. *Appl. Environ. Microbiol.* 71:2095-2105.
