# Supplementary material for: Development of Shuttle Vectors for Transformation of Diverse Rickettsia Species
Source: PLoS One. 2011 Dec 21;6(12):e29511. doi: 10.1371/journal.pone.0029511 (PMC3244465; doi:10.1371/journal.pone.0029511)
Supplement: Table S3 — Real-time PCR Amplification Primers to Determine Relative Copy Number of Native and Shuttle Vector-Transformed Rickettsia spp . (DOC) [file pone.0029511.s005.doc]

**Table S3. Real-time PCR Amplification Primers to Determine Relative Copy Number of Native and Shuttle Vector-Transformed *Rickettsia spp***.

| **Primer** | **Sequence (5’ to 3’)** | **Reference** |
| --- | --- | --- |
| qCSF | TCGCAAATGTTCACGGTACTTT | (1) |
| qCSR | TCGTGCATTTCTTTCCATTGTG | (1) |
| AcPa qCSR | TCGTACATTTCTTTCCATTGTGC | (2) |
| Rbellii qCSF | TCATGCATCTCTTTCCATTGTGC | (2) |
| Rbellii qCSR | CCGCAGATGTTCACAGTGCTTT | (2) |
| qGFPuvF | CAGTGGAGAGGGTGAAGGTGATGC | This publication |
| qGFPuvR | ACCATAAGAGAAAGTAGTGACAAGTGTTGGC | This publication |
| qHsp2F | GTAAACTAATAGAGCGGGAGAAAG | (2) |
| qHsp2R | TGAGGGCAAAAATGAACAATC | (2) |
| AcPa qHsp2F1 | GTAAACTAATAGAGCGAGCAAAAG | (2) |
| AcPa qHsp2R1 | TGAGGGCAAAAAGGAACAATC | (2) |
| qSpoTF2 | tgcaaatgtgatGCAGAACCAGAC | This publication |
| qSpoTR1 | AGATTTACGGAAGCTAGAAGTACAAATACG | This publication |
| pRAM23 qParAF | TAATGCACCAGCAACATCCTCTGC | This publication |
| pRAM23 qParAR | TCAGTGATTGCCGAGCCTGAAGAA | This publication |
| pRAM32 qParAF2 | ATCACAATTGCCAGCACAAAGGGC | This publication |
| pRAM32 qParAR2 | GCCTTGAGCGTCAGCATCCAATAA | This publication |

1. Stenos J, Graves SR, & Unsworth NB (2005) A highly sensitive and specific real-time PCR assay for the detection of spotted fever and typhus group rickettsiae. *Am. J. Trop. med. Hyg.* 73:1083-1085.

2. Baldridge GD*, et al.* (2010) Wide dispersal and possible mutliple origins of low-copy-number plasmids in *Rickettsia* species associated with blood-feeding arthropods. *Appl. Environ. Microbiol.* 76:1718-1731.
