# Supplementary material for: Development of Shuttle Vectors for Transformation of Diverse Rickettsia Species
Source: PLoS One. 2011 Dec 21;6(12):e29511. doi: 10.1371/journal.pone.0029511 (PMC3244465; doi:10.1371/journal.pone.0029511)
Supplement: Materials and Methods S1 — (DOC) [file pone.0029511.s006.doc]

**Supplementary Materials and Methods S1**

**Cloning and sequencing of *R. amblyommii* AaR/SC plasmid pRAM32.** All cloning and sequencing steps followed protocols published by the manufacturers unless stated otherwise. All primers were purchased from IDT (Coralville, IA), and all enzymes used were from either Promega (Madison, WI) or New England Biolabs (Ipswich, MA). A 15,043 bp fragment of the putative third plasmid of *R. amblyommii* AaR/SC (pRAM32) was obtained from a pJazz (Lucigen, Middleton, WI) clone as previously described [1]. It was sequenced on both strands using a primer walking strategy, and PCR primers targeting the ends of the cloned plasmid fragment (L13A, 5’- GAA GGG GAT AAT CCG ACC AT -3’ and L13B, 5’-TAA AGG CAT AGC GGA ATT GG-3’) were used to amplify the remaining portion of the plasmid. PCR was performed with system 3 of the Expand Long Template PCR system (Roche, Indianapolis, IN) using 100 ng of *R. amblyommii* AaR/SC genomic DNA as template. Cycling parameters were 1 cycle at 94^o^C for 2 min, 10 cycles at 94^o^C for 10 s, 48^o^C for 30 s and 68^o^C for 8 min, 25 cycles at 94^o^C for 15 s, 48^o^C for 30 s and 68^o^C for 8 min + 20 s per cycle and 1 cycle at 68^o^C for 7 min. The resulting 18,408 bp amplicon was gel purified with Freeze N Squeeze (Bio-Rad, Hercules, CA), concentrated by precipitation in 100% ethanol and digested with EcoRI, HindIII or XbaI. Individual fragments, isolated from a gel using the Zymoclean Gel DNA Recovery Kit (Zymo Research, Irvine, CA), were cloned into pGEM-3Z (Promega, Madison, WI), and DNA from individual colonies was prepared with the High Pure Plasmid Isolation kit (Roche). Three clones from each fragment were sequenced in forward and reverse orientation on an ABI 377 automated sequencer (Advanced Genetic Analysis Center, University of Minnesota) by a primer walking strategy beginning with pGEM primers M13F and M13R. Sequences were assembled using Sequencher v4.8 (Gene Codes Corporation, Ann Arbor, MI) and edited in MacVector 10.6.0 (Cary, NC). Edited sequence was prepared for submission to GenBank using Sequin Application Version 10.3 (NCBI) and annotated manually after submission to the NCBI Prokaryotic Genomes Automatic Annotation Pipeline (PGAAP). The nucleotide sequence of plasmid pRAM32 from *R. amblyommii* AaR/SC has been deposited in GenBank under the accession number CP002642.

**Construction of rickettsial shuttle vectors.** Constructs were derived from two *R. amblyommii* AaR/SC plasmids: pRAM18 (Figure S1) and pRAM32 (Figure S2).

**1)** **pRAM18 based shuttle vectors.** The complete pRAM18 plasmid (Figure S1A) [1] was released from the pJazz vector (Lucigen) with NotI (Figure S2B) and cloned into pSMART v2.0 BAC (Lucigen). The resulting construct pRAM18 v2.0 BAC (Figure S1C) was used as the starting material for construction of pRAM18/Rif/GFPuv (Figure S1D).

pRAM18/Rif/GFPuv construction. The 1.6 kbp cassette from pMW1650 (kindly provided by D. H. Wood, University of South Alabama) [2] containing the *R. prowazekii arr-2* rifampin resistance gene (*rpsLp-arr-2_Rp_*, or Rif) and a gene coding for green fluorescent protein driven by the rickettsial *ompA* promoter (*ompAp*-GFPuv, or GFPuv) (Figure S1E) was amplified with DW1011 and DW1013 primers [2] modified to contain ApaI restriction sites (ApaI DW1011 5’- gcg cgg ggc cct agg aac ata ctt gct ttt ata gg-3’;

ApaI DW1013 5’-TAT ATG GGC CCG TGT CTA GAG TCG ACC TGC AGG C-3’). Amplification reactions used *PfuTurbo* Hotstart DNA polymerase (Stratagene, La Jolla, CA) with 1 cycle at 95^o^C for 1 min, 30 cycles at 95^o^C for 1 min, 60^o^C for 1 min and 72^o^C for 3 min, and 1 cycle at 72^o^C for 10 min. The resulting amplicon was cloned into pCR4 (not shown) with the TOPO TA Cloning kit (Invitrogen, Carlsbad, CA) and clones were sequenced to confirm the integrity of the amplicon. The 1.6 kbp Rif/GFPuv cassette was isolated from a sequenced clone by digestion with ApaI and cloned into ApaI-digested pRAM18 v2.0 BAC. ApaI excises a 2.1 kbp fragment of v2.0 BAC containing the chloramphenicol resistance gene from pRAM18 v2.0 BAC, thus a 3-way ligation was used to restore v2.0 BAC components. pRAM18dRG (Figure S1F) and pRAM18dRGA (Figure S1G) were subsequently derived from the resulting construct pRAM18/Rif/GFPuv.

pRAM18dRG construction**.** A 7,111 bp fragment containing 5,445 bp of v2.0 BAC and the 1.6 kbp Rif/GFPuv expression cassette was released from pRAM18/Rif/GFPuv with NotI, blunt end repaired and dephosphorylated. A 5,879 bp fragment of pRAM18/Rif/GFPuv containing the *parA* and *dnaA*-like genes was recovered from a BbvCI digest by gel purification, blunt end repaired and cloned into the dephosphorylated, blunt end repaired 7,111 bp fragment of v2.0 BAC. The resulting 12,994 bp construct was designated pRAM18dRG.

pRAM18dRGA construction. The 1.6 kbp Rif/GFPuv cassette from pMW1650 was amplified with PstI-DW1011 (5’-GGG CTT CTG CAG CGG CCG CAA CAT ACT TGC TTT TAT AGG-3’) and ApaI-DW1013 primers as noted above except at a 64^o^C annealing temperature. The resulting amplicon was cloned into pCR4 as noted above, then released from pCR4 by digestion with PstI and the resulting Rif/GFPuv cassette was cloned into PstI-cut pGEM-3Z [NdeI knock-out] (Promega) yielding a pGEM vector containing *rif/gfp_uv_* (pGEM-RGA) (Figure S1H). The blunt end repaired 5,879 bp BbvCI fragment from pRAM18/Rif/GFPuv including the *parA* and *dnaA*-like genes*,* was generated as noted in preparation of pRAM18dRG, and then cloned into SmaI-cut pGEM-RGA. This 10,248 bp construct was designated pRAM18dRGA.

pRAM18dRGA[MCS] construction. A multiple cloning site (MCS) was prepared for insertion into pRAM18dRGA by hybridizing oligos encoding unique restriction sites and KpnI linkers. Briefly, equal quantities of forward and reverse PAGE- purified oligos (Forward: 5’- CGG ATT GGT ACC TAA GGA CAT AGT CGC TAG CAA GCG GCC GCA TTT AAA TCC GCG GCC TAG GTC CCG GGT ACC GAT CG-3’ and Reverse: 5’- CGA TCG GTA CCC GGG ACC TAG GCC GCG GAT TTA AAT GCG GCC GCT TGC TAG CGA CTA TGT CCT TAG GTA CCA ATC CG-3”) were denatured at 94°C for 2 min in 10 mM NaCl, cooled slowly to room temperature, column-purified (DNA Clean and Concentrator-25 kit, Zymo Research), digested with KpnI, gel-purified (Zymoclean gel recovery kit, Zymo Research), and ligated into dephosphorylated, KpnI-cut pRAM18dRGA. Clones were sequenced to ensure the integrity of the MCS.

pRAM18dRGA[AmTrCh] construction. An AmTr/mCherry cassette was inserted into pRAM18dRGA[MCS] at the AvrII restriction site. Specifically, a 948 bp cassette containing the *Anaplasma marginale* promoter *tr*, driving expression of *mCherry* [3,4,5] encoding a red fluorescent protein, was PCR-amplified with primers containing AvrII linkers (AmTrChF 5’-TAT ATC CTA GGC TCC TCC TAG AAC GAT CG-3’; AmTrChR 5’-GTC TCC CTA GGC TAT TTG TAT AAT TCG TCC ATT-3’) using conditions described for ApaI DW1011/DW1013 primers and a 56°C annealing temperature. The resulting amplicon was gel purified, digested with AvrII and ligated into dephosphorylated, AvrII-digested pRAM18dRGA[MCS].

**2) Construction of pRAM32dRGA, a pRAM32 shuttle vector.** pRAM32 (Figure S2A) from *R. amblyommii* AaR/SC genomic DNA was digested with SwaI and a 15,043 bp fragment was ligated into pJazz (Lucigen) [1] yielding pRAM32 SwaI frag 1 pJazz (Figure S2B). It was cut with XbaI and the XbaI fragments were ligated into pUC19. A clone containing the *parA* and *dnaA*-like genes of pRAM32 (pRAM32 XbaI frag 3) (Figure S2C) was identified, digested with PstI and ligated to the PstI-cut Rif/GFPuv cassette (generated with primers PstI-DW1011 and ApaI-DW1013; see preparation of pRAM18dRGA above) to form the 8,872 bp construct pRAM32dRGA (Figure S2E).

Constructs were verified to produce green fluorescence in *E. coli* by uv light microscopy using a Nikon Diaphot inverted microscope fitted with a Sapphire GFP filter or with a Nikon Eclipse E400 upright microscope (Nikon, Melville, NY) equipped with a FITC filter. All junctions were sequenced to determine orientation and integrity of ligated fragments. Each of the above constructs was prepared as an endotoxin-free plasmid maxi prep (Qiagen, Valencia, CA) for electroporation of rickettsiae.

**Digoxigenin-labeled probes.** *hsp2*, *gfp_uv_*, pRAM18 DNA Invertase gene and pRAM32 *recD* probes labeled with digoxigenin were synthesized with the PCR DIG Probe Synthesis kit (Roche, Indianapolis, IN). *hsp2* and *gfp_uv_* probes were synthesized as previously described [6,7]. The pRAM18 DNA Invertase gene and pRAM32 *recD* were amplified with 5 ng of plasmid template using primers pRAM18 DNA Invertase F (5’-TGC TCT CGC TTC TCT TTC AAC -3’) and pRAM18 DNA Invertase R (5’-GTT CAT CAG GAC AGT CGC TAA G-3’) or pRAM32 RecD F (5’-TGG TTT TTC CTG TTC CTG GTC C-3’) and pRAM32 RecD R (5’-TCA GAT GGT CAT TGT GGC TTA CC-3’) respectively, with cycling parameters of 1 cycle at 95^o^C for 2 min, 40 cycles at 95^o^C for 30 s, 50^o^C (pRAM18 DNA Invertase gene) or 52^o^C (pRAM32 *recD*) for 30 s and 72^o^C for 1 min and 1 cycle of 72^o^C for 7 min in a Robocycler (Stratagene).

**PFGE and Southern blot analyses.** Purified rickettsiae were embedded in agarose and lysed in situ by treatment with proteinase K, sodium lauryl sarcosine and 0.5 M EDTA. Intact DNA was separated by pulsed-field gel electrophoresis (PFGE) on a Beckman Geneline transverse alternating field electrophoresis system as previously described [1], or on a Chef Mapper XA System (Bio-Rad) with 0.5 X TBE using the Auto setting in CHEF mode. Chef Mapper XA System parameters were set as follows: DNA size range of 10 kbp to 100 kbp, a gradient of 6 V/cm, an angle of 120, a linear ramping factor, a calibration factor of 1, an initial switch time of 0.47 s and a final switch time of 8.53 s. The total run time was 20.18 h. Pulsed-field gels were depurinated and transferred onto Zeta Probe GT genomic membrane (Bio-Rad). The blots were hybridized at 55^o^C overnight with digoxigenin–labeled *hsp2*, *gfp_uv_*, pRAM18 DNA Invertase gene, or pRAM32 *recD* probes, washed at 55^o^C, labeled with anti-digoxigenin-AP Fab fragments (Roche) and detected with CDP-Star (Roche). Blots were exposed to Kodak X-OMAT AR film, or fluorescence from blots was captured with the Infinity 3 camera with Infinity Analyze Software version 5.0 (Lumenera Corporation, Ottawa, ON). Membranes to be re-used were stripped by rinsing briefly with Milli-Q water, washing two times at 37^o^C with 0.2 M sodium hydroxide/0.1 % SDS for 15 min and rinsing 5 min at room temperature in 2 X SSC. Before re-hybridization with a new probe, absence of prior signal was verified by adding detection reagent and re-exposure of membranes to film.

**Determination of shuttle vector and native plasmid copy numbers.** Real-time quantitative PCR (qPCR) and the relative quantification method [8,9] were used to determine shuttle vector and native plasmid copy number. Species-specific plasmids were used to generate standard curves with primers designed to target specific single copy genes: *gfp_uv_* for shuttle vectors, *hsp2* (pRM), *spoT* (pRAM18), pRAM23 *parA* (pRAM23) and pRAM32 *parA* (pRAM32) for native plasmids, and *gltA* for chromosomal DNA (Tables S2 and S3). qPCR was performed as previously described [1] in an Mx3005 qPCR cycler (Stratagene) with Brilliant II SYBR green qPCR master mix (Stratagene), 240 nM of each primer, serial dilutions of plasmid and rickettsial genomic DNAs adjusted to 10 ng DNA per sample with salmon sperm DNA (Promega). Cycling parameters to determine copy number for native pRM and shuttle vectors in transformed *Rickettsia* spp. were 1 cycle at 95^o^C for 10 min, 40 cycles of 95^o^C for 30 s, 58^o^C for 1 min and 72^o^C for 30 s, and a dissociation curve cycle of 95^o^C for 1 min, 54^o^C for 30 s and 95^o^C for 30 s to confirm product specificity. Parameters were identical when determining native plasmid copy numbers in *R. amblyommii* AaR/SC, except a 54^o^C annealing temperature was applied. MxPro version4 software was used to acquire the data and to reference genomic sample values to standard curves. Relative plasmid copy number per chromosomal equivalent was expressed as the mean amplification ratio of *gfp_uv_*/*gltA* (for shuttle vectors), *hsp2*/*gltA* (for native pRM), *spoT*/*gltA* (for native pRAM18), pRAM23*parA*/*gltA* (for native pRAM23) or pRAM32*parA*/*gltA* (for native pRAM32). Values for native pRM in *R. monacensis* shuttle-vector transformants (subculture 1 following transformation) and all shuttle vector transformants, were expressed as an average of amplification from three separate plates with all samples run in triplicate. Determination of native pRM in *R. monacensis* pRAM18dRGA-transformants at subcultures 8 and 15, and calculations for the copy number of individual plasmids in *R. amblyommii* were based on the average of triplicate samples from one plate.

**Supplemental References Cited**

1. Baldridge GD, Burkhardt N, Labruna MB, Pacheco RC, Paddock CD, et al. (2010) Wide dispersal and possible multiple origins of low-copy-number plasmids in *Rickettsia* species associated with blood-feeding arthropods. Appl Environ Microbiol 76: 1718-1731.

2. Liu Z-M, Tucker AM, Driskell LO, Wood DO (2007) *mariner-*based transposon mutagenesis of *Rickettsia prowazekii*. Appl Environ Microbiol 73: 6644-6649.

3. Barbet AF, Agnes JT, Moreland AL, Lundgren AM, Alleman AR, et al. (2005) Identification of functional promoters in the msp2 expression loci of *Anaplasma marginale* and *Anaplasma phagocytophilum*. Gene 149: 89-97.

4. Felsheim RF, Herron MJ, Nelson CM, Burkhardt N, Barbet AF, et al. (2006) Transformation of *Anaplasma phagocytophilum*. BMC Biotechnol 6: 42. doi:10.1186/1472-6750-1186-1142.

5. Shaner NC, Campbell RE, Steinbach PA, Giepmans BNG, Palmer AE, et al. (2004) Improved monomeric red, orange and yellow fluorescent proteins derived from *Discosoma* sp. red fluorescent protein. Nat Biotechnol 22: 1567-1572.

6. Baldridge GD, Burkhardt N, Herron MJ, Kurtti TJ, Munderloh UG (2005) Analysis of fluorescent protein expression in transformants of *Rickettsia monacensis*, an obligate intracellular tick symbiont. Appl Environ Microbiol 71: 2095-2105.

7. Baldridge GD, Burkhardt NY, Felsheim RF, Kurtti TJ, Munderloh UG (2007) Transposon insertion reveals pRM, a plasmid of *Rickettsia monacensis*. Appl Environ Microbiol 73: 4984-4995.

8. Lee C, J. K, Shin SG, Hwang S (2006) Absolute and relative QPCR quantification of plasmid copy number in *Escherichia coli*. J Biotechnol 123: 273-280.

9. Livak KJ, Schmittgen TD (2001) Analysis of relative gene expression data using real-time quantitative PCR and the 2∆∆–CT method. Methods 25: 402-408.

10. Stenos J, Graves SR, Unsworth NB (2005) A highly sensitive and specific real-time PCR assay for the detection of spotted fever and typhus group rickettsiae. Am J Trop med Hyg 73: 1083-1085.
